# Estimating aman and aus rice area in Bangladesh using sentinel-1 imagery and machine learning algorithms

**DOI:** 10.1371/journal.pone.0355502

**Published:** 2026-09-18

**Authors:** H. M. Hamidur Rahman, Hasan Mahmud, Md Mahfuzul Hasan, S. G. Hussain

**Affiliations:** 1 Computer and GIS Unit, Bangladesh Agricultural Research Council, Dhaka, Bangladesh; 2 ACASA Project, Bangladesh Agricultural Research Council, Dhaka, Bangladesh; 3 Center for Environmental and Geographic Information Services, Public Trust, Ministry of Water Resources, and Bangladesh Agricultural Research Council, Dhaka, Bangladesh; Van Lang University: Truong Dai hoc Van Lang, VIETNAM

## Abstract

Rice (*Oryza sativa L.*) is Bangladesh’s staple crop, cultivated across three distinct seasons: Rabi (Boro), Kharif-1 (Aus) and Kharif-2 (Aman). This study develops an integrated, cloud-independent framework for mapping Aus and Aman rice areas using freely available Sentinel-1 C-band Synthetic Aperture Radar (SAR) imagery; to accomplish this, supervised machine-learning (ML) algorithms are used in the Google Earth Engine (GEE) platform. Time-series dual-polarization (VV and VH) backscatter data were analyzed for June–November 2021 (Aman) and March–August 2022 (Aus). Four ML models such as Classification and Regression Tree (CART), Random Forest (RF), k-Nearest Neighbour (k-NN), and Support Vector Machine (SVM) were evaluated to identify the most accurate mapping approach. Among all ML models, k-NN achieved the highest accuracy of 94.5% and 99.8% for Aman and Aus, respectively. Despite the higher accuracy, RF for Aman (92.7% accuracy) and CART for Aus (99.6%) were finally selected based on their lower deviation from area estimates reported by Bangladesh Bureau of Statistics (BBS). The estimated Aman area (5.72 Mha) was closely matched with the official figure from the Bangladesh Bureau of Statistics (BBS), while the Aus estimate (0.76 Mha) showed a 29% underestimation compared to BBS data (1.06 Mha). However, the results demonstrate that Sentinel-1 SAR combined with ML classifiers provides a reliable, scalable, and weather-resilient approach for multi-season rice mapping. The proposed methodology establishes a strong foundation for timely production forecasting and climate-resilient agricultural planning, particularly in cloud-prone tropical regions.

## 1. Introduction

More than half of the world’s population relies on rice (*Oryza sativa L.*) as a primary staple food, and over 90% of production is concentrated in Asia’s humid and cloud-prone regions. In Bangladesh, rice dominates the agricultural landscape, covering approximately 8.83 million hectares (Mha) of cultivable land and providing livelihoods for nearly 45% of the population. Aus (Kharif-I; March–August), Aman (Kharif-II; July–December), and Boro (Rabi; December–May) are the three main rice growing seasons. Together, the Aman and Aus seasons form the foundation of the nation’s rainfed rice system. Of the total rice area, roughly 5.73 Mha is cultivated during Kharif-2 and 1.06 Mha during Kharif-1 [1]. Aman rice benefits from monsoon rains and extensive flooding, whereas Aus is sown earlier, relying on pre-monsoon precipitation or supplementary irrigation. This seasonal diversity reflects Bangladesh’s complex hydrological and climatic regime, yet it also exposes rice production to heightened risks from rapid urbanization, industrial expansion, and increasingly erratic rainfall driven by climate change.

Accurate and timely estimation of rice-growing areas across both Aus and Aman seasons is crucial for yield forecasting, procurement planning, and food-security assessments. Traditional field surveys, while reliable at local scales, are labor-intensive, expensive, and often unable to deliver near-real-time information at the national level. This limitation is particularly acute for Aman and Aus rice, as persistent cloud cover and frequent flooding often render optical observations unusable during the peak growing period. Consequently, cloud-independent remote sensing approaches are needed to ensure consistent monitoring of seasonal rice dynamics in monsoon, fragmented land structural and high cropping intensive environments.

Synthetic Aperture Radar (SAR) offers a powerful alternative for agricultural monitoring. Operating at C-band, Sentinel-1 SAR provides weather and illumination-independent observations of surface backscatter, which is highly sensitive to variations in water, soil moisture, and canopy structure. Its dual-polarization (VV and VH) configuration and 12-day revisit cycle enable detailed temporal tracking of key agronomic operations and phenological transitions such as puddling, transplanting, tillering, and heading [2,3]. In parallel, advances in machine learning (ML) have enabled robust classification of complex, non-linear backscatter signatures. Algorithms such as Decision Tree (DT), Random Forest (RF), k-Nearest Neighbour (k-NN), and Support Vector Machine (SVM), have consistently achieved 85–95% accuracy in distinguishing rice from other land-cover classes across monsoon-dominated ecosystems [4–6]. Nevertheless, most preceding applications have focused on a single season or region-specific analyses, leaving a gap in national-scale, cross-seasonal evaluations of SAR–ML frameworks against official agricultural statistics.

Optical satellite data such as MODIS NDVI, Landsat, and Sentinel-2 have been widely used for rice mapping but remain severely constrained by persistent cloud cover during the monsoon months. A combination of spatiotemporal data fusion and a phenology-based algorithm have shown promise in mapping paddy rice in cloudy regions. This approach utilizes a modified neighborhood similar pixel interpolator to remove clouds from Sentinel-2 and Landsat images, achieving an overall accuracy of 93% [7]. The flexible spatiotemporal data fusion model integrates MODIS and Sentinel-2 data, producing high-resolution vegetation indices that effectively capture rice growth stages. Likewise, the Auto-OSDF method employs decision-level fusion of optical and synthetic aperture radar (SAR) imagery, maintaining high accuracy (above 93%) even under cloud cover levels between 10% and 50% [8].

In India, a sample-independent mapping method using the Synthetic Aperture Radar-based Rice Index (SPRI) has been developed, achieving 10 m resolution maps despite frequent cloud cover [9]. This method effectively identifies rice planting locations by analyzing backscatter coefficients. Unlike optical sensors, Sentinel-1 can operate effectively in cloudy conditions, making it ideal for regions with persistent monsoon rain clouds [10,11].

Bangladesh presents an ideal testbed for developing and validating such a unified rice-mapping framework. The contrasting hydrological conditions of the Aus and Aman seasons provide a natural experiment for examining the seasonal transferability and robustness of SAR-based models under differing flood and moisture regimes. Building on this premise, the present study aims to develop and assess a Sentinel-1–based ML approach for the consistent, national-scale estimation of Aus and Aman rice areas in Bangladesh. By jointly analyzing Aus and Aman rice distributions, this study provides new insights into the spatio-temporal dynamics and climatic vulnerability of Bangladesh’s rice production system. Aman rice, being more susceptible to flooding, shows greater year-to-year variation, as demonstrated by the 2024 monsoon floods that damaged nearly 0.2 Mha of cropland [12]. Conversely, Aus cultivation though smaller in extent serves as an early indicator of monsoon onset and water stress. Integrating both seasons within a unified monitoring framework thus supports early warning, crop insurance, and disaster response mechanisms. Ultimately, the findings reinforce the strategic importance of Sentinel-1 SAR and ML for climate-resilient, near-real-time rice monitoring.

While SAR-based machine learning and deep learning approaches have been widely used for rice mapping, most existing studies focus on single-season analysis, regional scales, or rely on intensive training datasets. Few studies have evaluated the comparative performance of multiple machine learning algorithms for multi-season rice mapping at a national scale, particularly in highly fragmented and flood-prone agroecosystems such as Bangladesh. This study addresses this gap by implementing a unified SAR-based framework for both Aus and Aman seasons, comparing multiple ML classifiers under identical conditions, and evaluating not only classification accuracy but also consistency with official agricultural statistics (BBS).

## 2. Conceptual framework

This study utilizes Sentinel-1 Synthetic Aperture Radar (SAR) data to estimate Aus and Aman rice areas across Bangladesh through the Google Earth Engine (GEE) cloud-computing framework. The entire Bangladesh was selected as the Region of Interest (ROI) due to its diverse agroecological zones and the dominance of monsoon dependent rice cultivation. Two rice seasons: Kharif-I (Aus) and Kharif-II (Aman) were examined. Representative sample ground reference data were collected from varied rice-growing areas for each season to capture the spatio-temporal variability in radar backscatter responses. These samples formed the basis for deriving spectral–temporal signatures and defining classification thresholds to distinguish rice from other land-cover types.

The methodological workflow integrates Sentinel-1 pre-processing, temporal feature extraction, and ML-based classification within the GEE environment. Pre-processing steps include radiometric calibration, terrain correction, and speckle filtering to derive sigma-naught (σº) backscatter values in both VV and VH polarizations. As all these steps are inbuilt into the GEE platform, therefore, minimal manual intervention is needed. Different supervised classification algorithms were trained to map Aus and Aman rice distributions separately.

**Fig 1:**
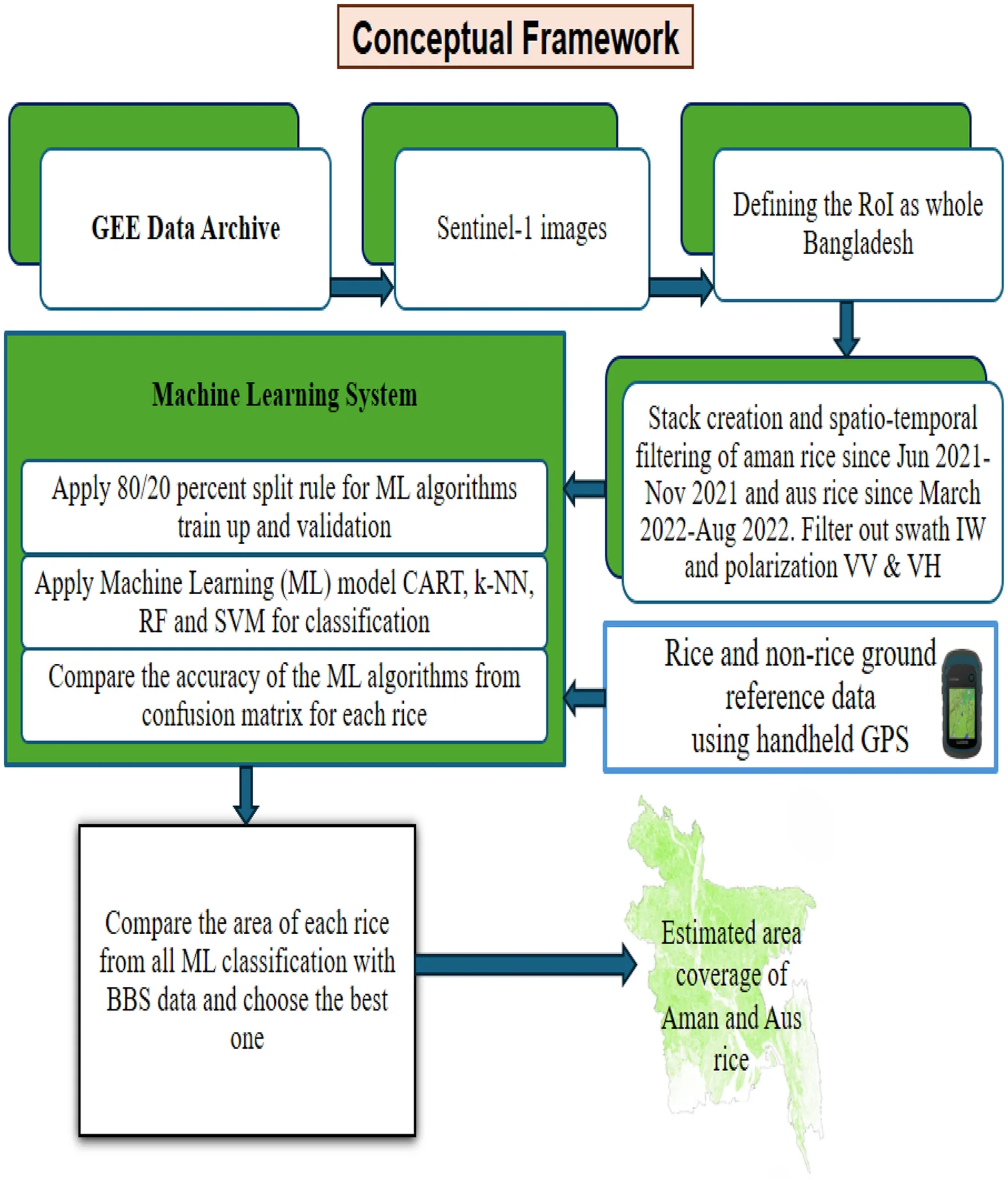
Conceptual framework of Aman and Aus rice area estimation (Source: Rahman and Hussain [14]).

In Bangladesh, agricultural planning and food security assessments rely on the official statistics published by the Bangladesh Bureau of Statistics (BBS). Therefore, BBS estimates were used as an independent benchmark to evaluate the classification results. Rice area estimates from each classifier were compared with BBS data, and the model with the closest agreement was selected to produce a national-scale map of Aman and Aus rice area across Bangladesh. The overall methodological framework is illustrated in Fig 1.

## 3. Materials and methods

### 3.1 Study area

Bangladesh lies between 20°34’-26°38’ North Latitude and 88°01’-92°41’ East Longitude, covering an area of approximately 147,570 km² in South Asia. It is bordered by India to the west, north, and east; Myanmar (Burma) on the southeast; and the Bay of Bengal on the south. Geographically, it is situated at the confluence of three of the major river systems, such as Ganges (Padma), Brahmaputra (Jamuna), and Meghna which together form the Ganges-Brahmaputra-Meghna (GBM) Delta, the largest deltaic plain on Earth. The country’s agroecological landscape is predominantly composed of low-lying floodplains intersected with numerous rivers. The climate is humid subtropical, characterized by three distinct seasons: a hot pre-monsoon, a monsoon marked by intense rainfall, and a cool dry winter. Annual precipitation varies significantly from roughly 1,400 mm in the northwest to over 4,000 mm in the northeast [13]. These climatic gradients, coupled with diverse soil and hydrological conditions, strongly influence rice cultivation patterns and seasonal variability in crop phenology across the country.

### 3.2 Study period and sample size

Sentinel-1 SAR imagery and corresponding ground-reference data were gathered for two cropping periods: Aman (Kharif-II; June–November 2021) and Aus (Kharif-I; March–August 2022) to assist seasonal rice area estimation. Field data collection employed a structured survey questionnaire designed to capture both quantitative and qualitative information on land use, cropping stage, and environmental context. A total of 344 training samples were obtained for Aman and 374 samples for Aus (Table 1) rice. Ground reference data of Aus is 73, which is minimal compared to Aman, because Aus is cultivated in very minimal area in Bangladesh. Further details on data collection and processing methods are available in Rahman and Hussain [14]. This study used publicly available satellite data and non-identifiable field observations.

**Table 1:** Sample distribution of ground reference data used in this study.

Crop Season	Duration	Rice	Non-Rice	Total
Aman (Kharif-2 Season)	June – November 2021	301	43	344
Aus (Kharif-1 Season)	March – August 2022	73	301	374

**Fig 2:**
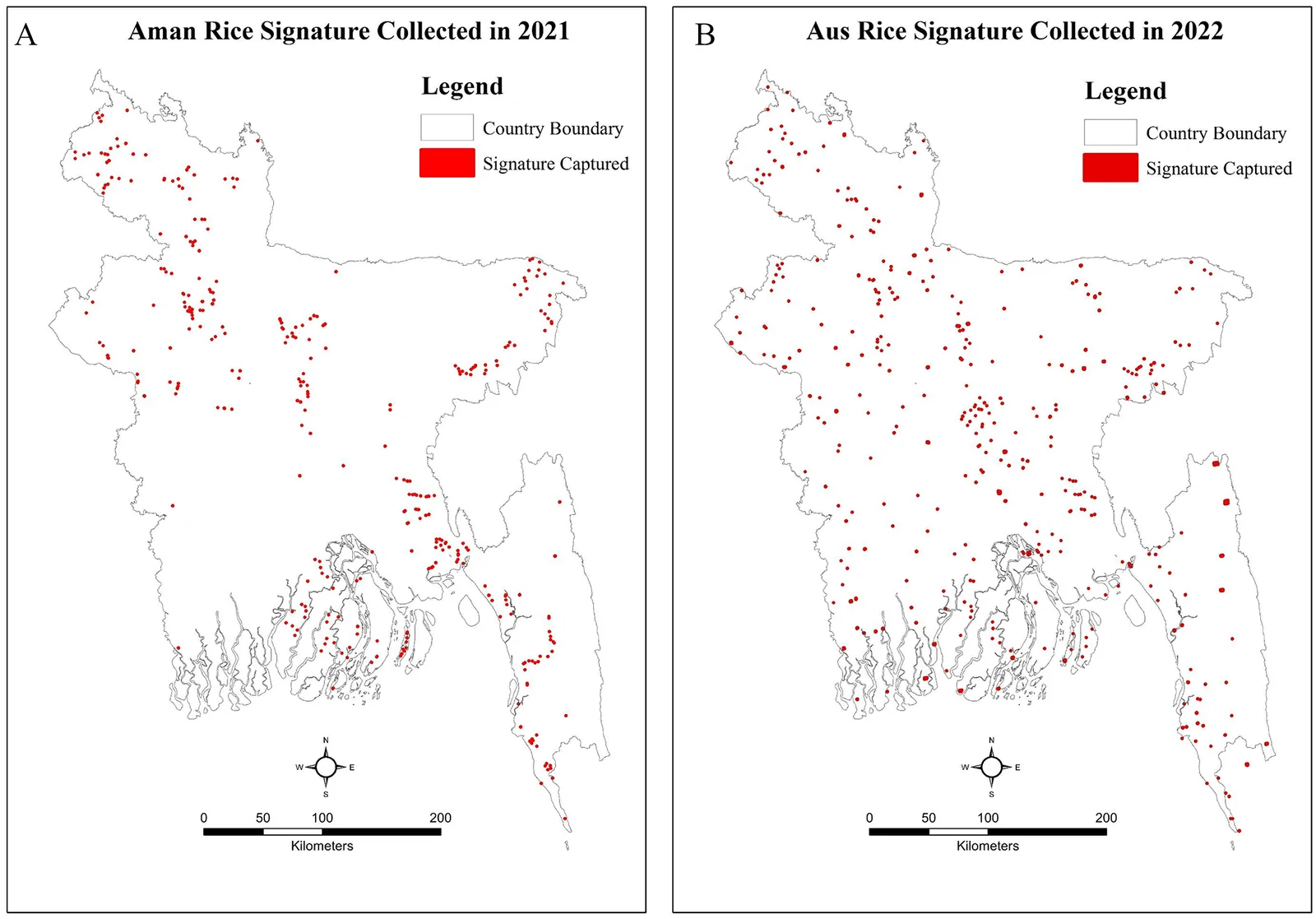
A. Ground reference data locations of Aman (Source: Boundary map of Bangladesh is collected from BARC website [22]). B. Ground reference data locations of Aus (Source: Boundary map of Bangladesh is collected from BARC website [22]).

Using regional agricultural statistics and visual interpretation of high-resolution Google Earth images, sample locations within high-density rice-cultivated zones were chosen. Other Non-Rice land-cover types, including water bodies, settlements, and other crops were also sampled in order to increase model generality and classification accuracy. The spatial distribution of the reference samples (Fig 2A and 2B) demonstrates comprehensive coverage across Bangladesh’s principal rice-growing regions. The broad geographic and environmental diversity captured by these samples provides an ideal testbed for evaluating the performance and transferability of Sentinel-1 SAR–based crop classification models under varying climatic and hydrological conditions. The geographical boundary shapefile of Bangladesh was obtained from the open-source Bangladesh Agricultural Research Council (BARC) website and used for further analysis, including spatial distribution and ground reference image generation.

### 3.3 Ground-truth data collection and pre-processing

The platform’s automated pre-processing chain, which includes radiometric calibration, terrain correction, orbit and incidence-angle normalization, and speckle filtering, was applied to all Sentinel-1 datasets processed within GEE. These procedures are integrated within the GEE Sentinel-1 collection and ensure that all images are radiometrically and geometrically consistent. As a result, no additional external pre-processing was required. This built in automation is a substantial advantage of GEE, allowing efficient, consistent, and large-scale handling of SAR datasets [15]. For each season, monthly median composites time series were generated for Aus (March–August). A similar Sentinel-1 approach for monitoring monsoon-season rice was demonstrated by Aziz et al. [16]. Finally, the rice areas estimate produced by each classifier were compared with official Bangladesh Bureau of Statistics (BBS) records for 2021–2022 (Aman) and 2022–2023 (Aus) to evaluate areal consistency and model reliability.

Ground-truth data were collected through field surveys and GPS-based observations to support classifier training and validation. Initially, 602 polygons for Aman rice and 374 polygons for Aus rice were digitized as geospatial shapefiles. Each sample was recorded using handheld GPS units with a positional accuracy of ±5m and categorized as either rice or Non-Rice (including water bodies, settlements, fallow land, and other crops).

### 3.4 Model training and calculation of matrices

The preliminary model trained on the original shapefile performed sub-optimally due to imbalanced polygon sizes. Non-Rice polygons were substantially larger than rice polygons, creating bias toward the Non-Rice class. To address this issue, polygon areas were standardized to approximately 100 m^2^ using a custom Python script developed in ArcMap Toolboxes. After removing the outliers and inconsistent samples for Aman rice, 344 high-quality polygons were retained for classification training. Alongside the spatial data, farmer interviews provided valuable supplementary information on rice variety (high-yielding vs. traditional), transplanting and harvesting dates, irrigation practices, fertilizer and pesticide use, and yield levels. The training datasets were labeled as rice (1) and Non-Rice (0), and pixel values corresponding to each training location were extracted from the stacked composites. Field polygons were *spilt* to ensure independence. Each model was trained using the full feature stack of VV and VH backscatter bands. These contextual attributes enhanced understanding of spatial and temporal patterns in rice cultivation and supported more accurate interpretation of classification outcomes. Classification performance was evaluated using the independent test samples, and standard accuracy metrics were computed, including Overall Accuracy (OA), Kappa coefficient, and class-specific User’s and Producer’s accuracies (Table 2). From this table, we see that, Kappa coefficient for Aman lies between 0.579 to 0.871 and for Aus 0.258 to 0.613. The 5-fold cross-validation results indicate stable model performance, while the low standard deviation values indicate that the model is robust and not sensitive to data partitioning.

**Table 2:** Confusion matrices and classification performance metrics for rice mapping using different machine learning methods during Aman and Aus rice.

Rice Season	Method	Class	Rice	Non-Rice	Precision	Recall	Kappa Coeff.	F1 Score	Overall Accuracy	CV Accuracy (Mean ± SD)
**Aman**	CART	Rice	350	46	0.90	0.88	0.64	0.75	0.85	0.869 ± 0.014
		Non-Rice	41	133	0.74	0.76		0.89		
	k-NN	Rice	384	12	0.95	0.97	0.87	0.91	0.95	0.925± 0.015
		Non-Rice	19	155	0.93	0.89		0.96		
	RF	Rice	374	22	0.94	0.94	0.81	0.87	0.93	0.921 ± 0.004
		Non-Rice	23	151	0.87	0.87		0.94		
	SVM	Rice	365	31	0.85	0.92	0.58	0.69	0.82	0.816± 0.015
		Non-Rice	65	109	0.78	0.63		0.88		
**Aus**	CART	Rice	29344	72	1.00	1.00	0.26	0.30	1.00	0.996± 0.0002
		Non-Rice	47	26	0.24	0.29		1.00		
	k-NN	Rice	29410	38	1.00	1.00	0.61	0.61	1.00	0.998± 0.0001
		Non-Rice	6	35	0.85	0.48		1.00		

**Note:** For **Aman** original polygons: 344; Pixels after *sampleRegions()*: 2680; Training pixels: 2110; Testing pixels: 570; for **Aus** original polygons No.: 374; Pixels after *sampleRegions()*: 147694; Training pixels: 118226; Testing pixels: 29468.

Due to computational limitations of the Google Earth Engine (GEE) free-tier environment, the classified raster for the entire study area (Bangladesh) was exported in 38 tiles and processed locally. The exported tiles were mosaicked in ArcMap to generate a seamless national-scale classification.

**Fig 3:**
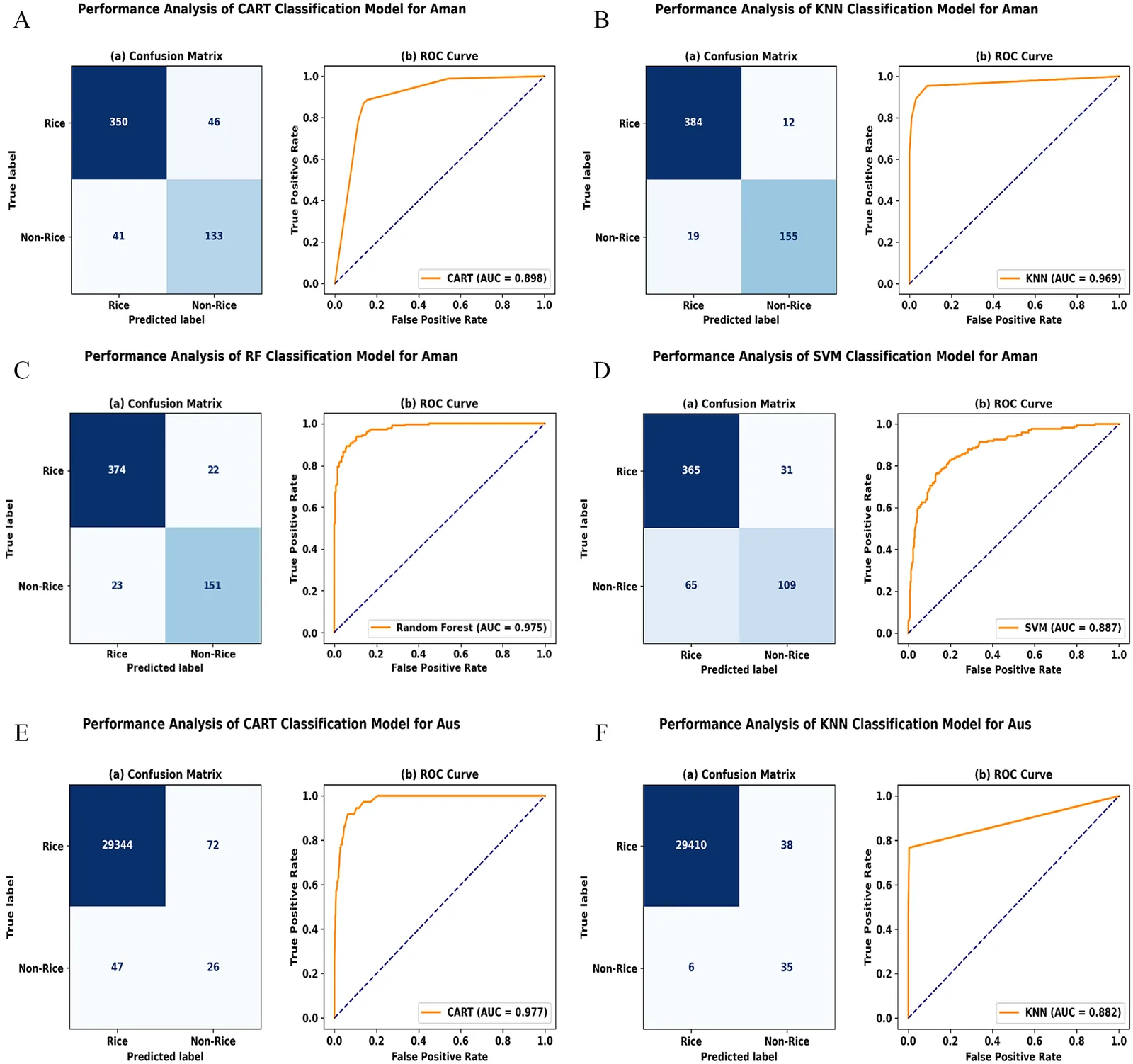
A. Performance analysis of CART classification model for Aman. B. Performance analysis of k-NN classification model for Aman. C. Performance analysis of RF classification model for Aman. D. Performance analysis of SVM classification model for Aman. E. Performance analysis of CART classification model for Aus. F. Performance analysis of k-NN classification model for Aus.

The classification performance was further evaluated using confusion matrices and Receiver Operating Characteristic (ROC) analysis. As depicted in the Fig 3A-3F, the ROC analysis confirms model discrimination ability, with RF and CART achieving the highest AUC values for Aman and Aus rice respectively. The ROC curve was generated using class probability outputs from the classifiers, and the Area Under the Curve (AUC) was computed to assess separability between rice and Non-Rice classes. The results demonstrate strong discriminatory capability of the model.

Rice area estimation was conducted in a projected coordinate reference system (ESRI:102954, Bangladesh Transverse Mercator 2010), ensuring accurate representation of ground distances and areas. The raster was maintained at a spatial resolution of 10 m × 10 m, corresponding to a pixel area of 100 m^2^. Total rice area was computed using attribute table–based pixel counting, where pixel counts were multiplied by the corresponding ground area per pixel.

### 3.5 Model configuration

A Random Forest classifier (ee.Classifier.smileRandomForest) was implemented using 100 trees. The number of variables per split was set to the square root of the total number of input features. The minimum leaf population was set to 1, and the bagging fraction was 0.5. No restriction was imposed on the maximum number of nodes per tree. In case of CART, this research used ee.Classifier.smileCart() with default values minLeafPopulation = 1, maxNodes = 1000, reduceErrorPruning = false, splitFraction = 0.999. No explicit tuning was applied. SVM methods used ee.Classifier.libsvm() with default Earth Engine values, where cost (C) = 10, gamma = auto (1 / number of features) and Nu, p, tolerance = defaults (Earth Engine internal). Like as k-NN was implemented with k = 5 using ee.Classifier.smileKNN.

## 4. Results

### 4.1 Spatial distribution of aman and aus rice

**Fig 4:**
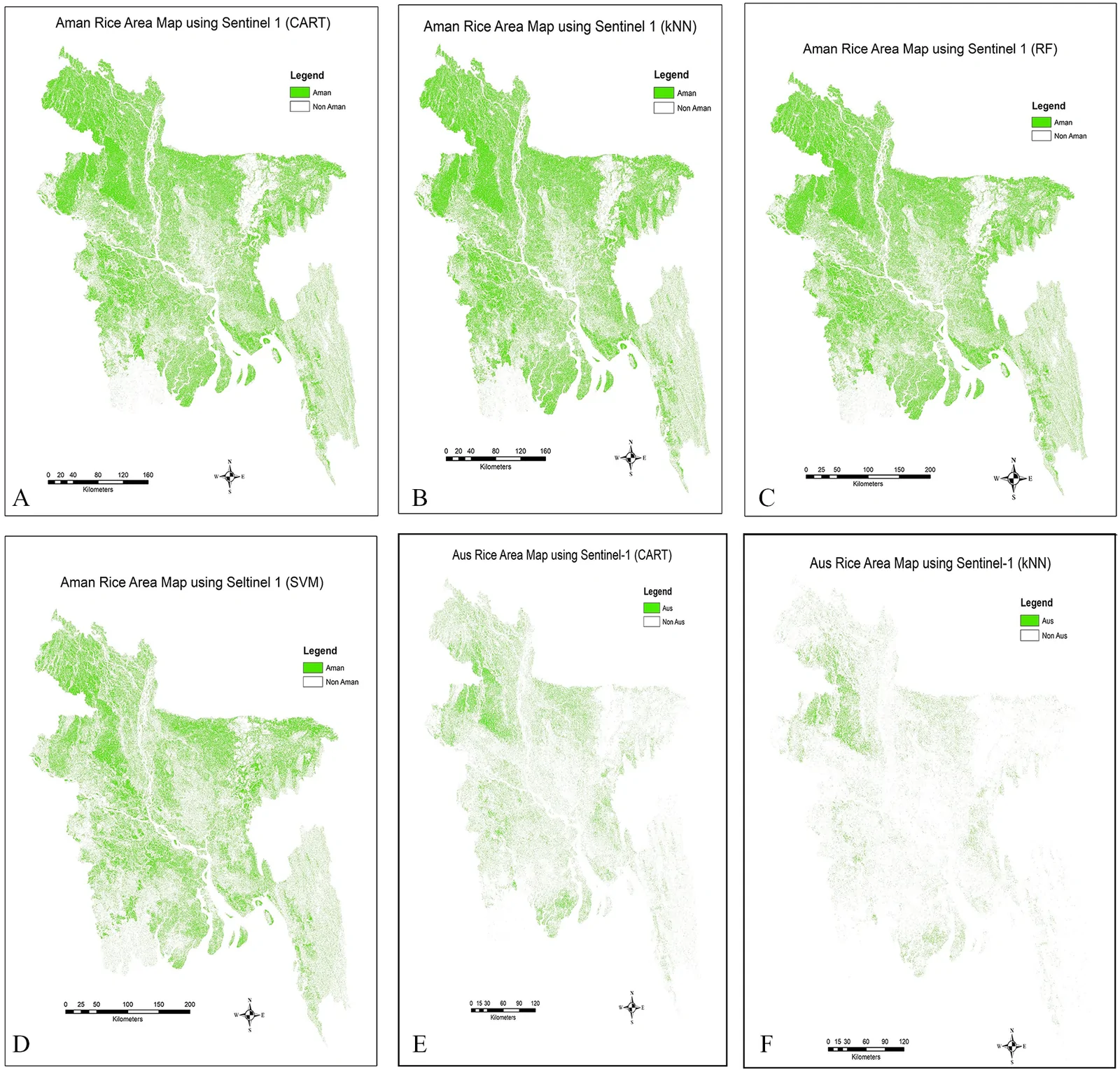
A. Spatial distribution of Aman (using CART) rice in Bangladesh (Source: Boundary map of Bangladesh is collected from BARC website [22]). B. Spatial distribution of Aman (using k-NN) rice in Bangladesh (Source: Boundary map of Bangladesh is collected from BARC website [22]). C. Spatial distribution of Aman (using RF) rice in Bangladesh (Source: Boundary map of Bangladesh is collected from BARC website [22]). D. Spatial distribution of Aman (using SVM) rice in Bangladesh (Source: Boundary map of Bangladesh is collected from BARC website [22]). E. Spatial distribution of Aus (using CART) rice in Bangladesh (Source: Boundary map of Bangladesh is collected from BARC website [22]). F. Spatial distribution of Aus (using k-NN) rice in Bangladesh (Source: Boundary map of Bangladesh is collected from BARC website [22]).

Using Sentinel-1 SAR images, the four ML classifiers CART, k-NN, RF, and SVM successfully identified the spatial distributions of Aman (June–November 2021) and CART and k-NN delineated for Aus (March–August 2022) separately across Bangladesh (Fig 4A-4F). In line with the nations monsoon dependent rice systems, Aman rice showed a wide and continuous distribution throughout the northern and central agroecological zones, especially in the division of Mymensingh, Rangpur, Rajshahi, and Dhaka. Due to flood damage and extended inundation, coastal and haor regions, such as Barishal and Sylhet division, displayed relatively lower density. In contrast, Aus rice was primarily concentrated in central and western floodplains notably Dhaka, Rajshahi, Faridpur, and Barishal divisions where early monsoon rainfall or supplemental irrigation enables pre-monsoon cultivation. Compared to Aman, Aus rice fields were more fragmented and covered smaller contiguous areas, reflecting the season’s shorter crop cycle and higher variability in rainfall onset. The radar VH backscatter time series effectively captured phenological transitions in both crops. Aman rice exhibited a pronounced increase in VH backscatter during the tillering and heading stages, followed by a gradual decline approaching harvest. Aus rice showed a similar pattern but with compressed timing, corresponding to its shorter growth cycle.

### 4.2 Temporal VV/VH backscatter profiles

**Fig 5:**
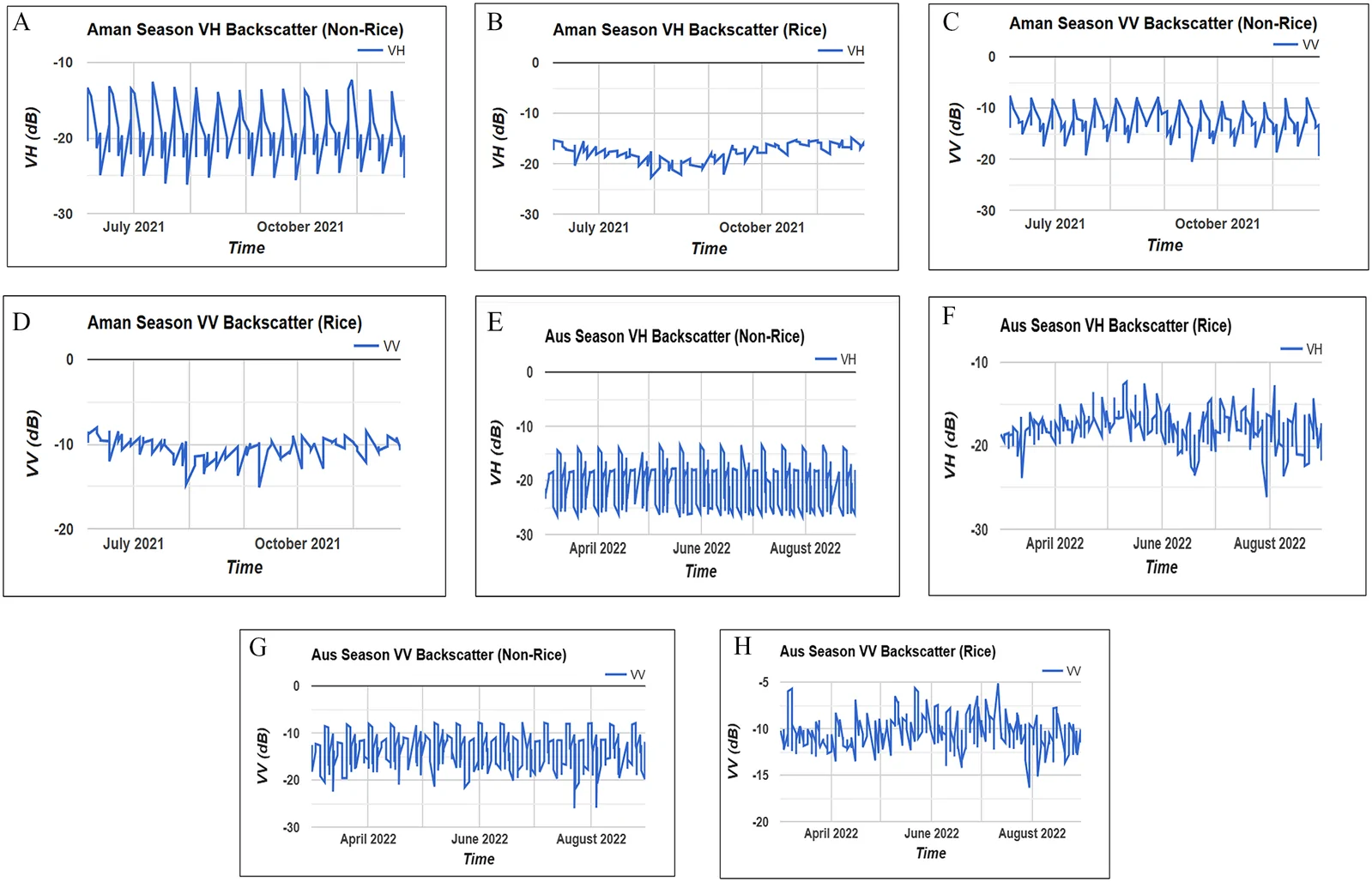
A. Temporal VH profiles of Aman season Non-Rice classes. B. Temporal VH profiles of Aman season Rice Classes. C. Temporal VV profiles of Aman season Non-Rice classes. D. Temporal VV profiles of Aman season rice classes. E. Temporal VH profiles of Aus season Non-Rice classes. F. Temporal VH profiles of Aus season Rice classes. G. Temporal VV profiles of Aus season Non-Rice classes. H. Temporal VV profiles of Aus season Rice classes.

The temporal evolution of VV and VH backscatter for rice and Non-Rice classes are shown in Fig 5A-5H. Rice fields exhibit a characteristic temporal signature, with low backscatter during flooding/transplanting followed by a gradual increase during vegetative growth and a decline near harvest. This temporal separability justifies the use of time-series SAR features for classification.

### 4.3 Area estimation through machine learning

For Aman rice, the RF model and k-NN achieved the highest overall accuracy (92.68% and 94.56%) but the deviation with BBS statistics is 0.01% and 7% respectively. while CART and SVM were less consistent due to misclassification of rice areas. For Aus rice, the accuracy obtained by the k-NN (99.80%) and CART (99.57%). Though RF yields better results in Aman area estimation but in the case of Aus it produced very poor results (Table 3). Similar result found for SVM also. Therefore, the RF and SVM are avoided for Aus area delineation. The high accuracies for Aus reflect clearer phenological signals during its relatively cloud-free growing season and reduced spectral confusion with other crops and we considered CART algorithm for better results.

**Table 3:** Comparative accuracy and area estimation of Aman rice using four ML classifiers applied to Sentinel-1 SAR imagery with BBS (Fiscal year 2021−22 for Aman and Fiscal Year 2022−23 for Aus).

Rice	Classification Algorithm	Pixel count (10m*10m)	Estimated Area (Million Ha)	BBS Area (Million Ha)	Classification Accuracy of Algorithm (%)	Area Deviation from BBS (%)
Aman	CART	515,641,129	5.16	5.72	84.73	−10.00
	k-NN	531,116,050	5.31		94.56	−7.00
	RF	571,969,367	5.72		92.68	+0.01
	SVM	388,188,188	3.88		82.14	−32.00
Aus	CART	76,349,280	0.76	1.06	99.57	−28.05
	k-NN	69,252,384	0.69		99.80	−34.73

**Fig 6:**
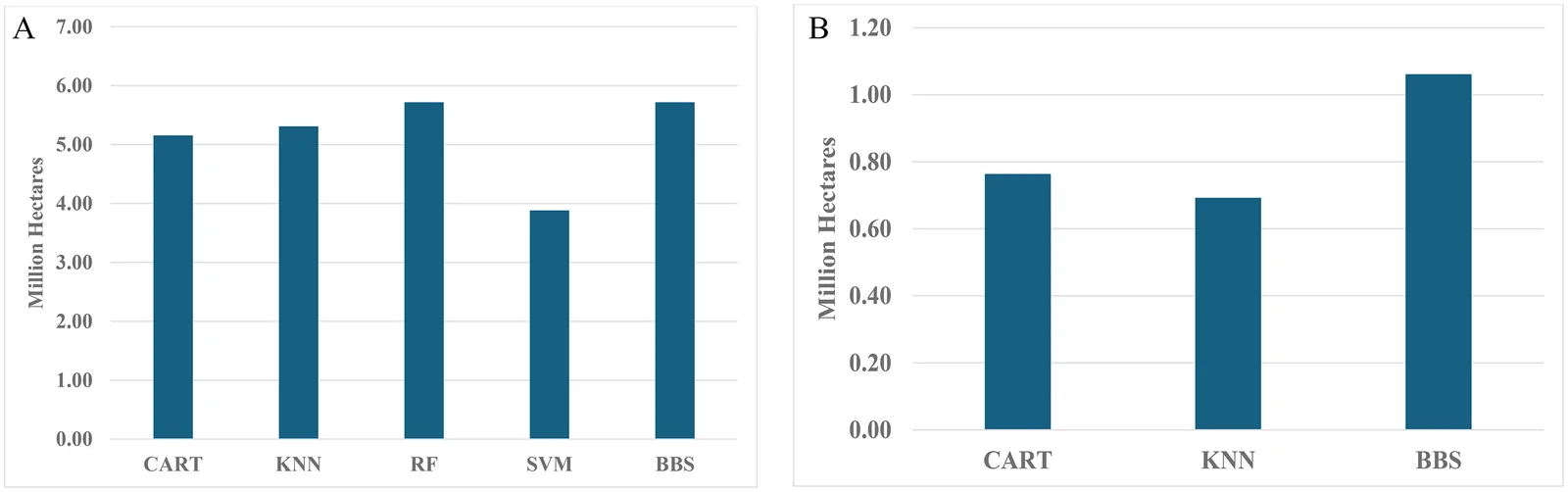
A. Comparison between the algorithm and the BBS output of estimated Aman area. B. Comparison between the algorithm and the BBS output of estimated Aus area.

The RF model’s ensemble averaging mechanism mitigated local misclassifications caused by heterogeneous land use [17], making it robust for fragmented smallholder landscapes typical of Bangladesh. Visual inspection also confirmed improved delineation of paddy clusters and suppression of false positives along water bodies. Its ensemble averaging approach mitigated the effects of localized noise in the SAR signal, particularly in flood-prone areas. Fig 6A shows RF estimates align with BBS data for Aman and CART results shows comparatively lower deviation with BBS than other methods for Aus (Fig 6B).

## 5. Discussion

In this study, overall categorization accuracy for Aman rice ranged from 82% to 94%, which is in line with reports for similar monsoon ecosystems in South and Southeast Asia, where Sentinel-1-based rice mapping achieved accuracies between 85% and 93% [4,5]. These findings demonstrate that Sentinel-1 SAR may effectively complement optical sensors for operational crop monitoring under persistently cloudy monsoon conditions and support the resilience of the established procedure.

The effectiveness of Sentinel-1 data for rice mapping and phenological monitoring is further supported by earlier research. For example, Raman et al. [18] reported 91.5% overall accuracy (κ = 0.83) in Tamil Nadu, India, while Phung et al. [19] found a strong correlation between Sentinel-1-derived growth indicators and in-situ observations (R² = 0.92; RMSE = 7.3 days). Although optical sensors such as Sentinel-2 have produced slightly higher classification accuracies for Boro rice (94.1% OA; Rahman & Hussain [14],), their effectiveness during the Aman season remains constrained due to persistent cloud cover. In contrast, the current findings demonstrate that Sentinel-1 is suitable for all weather and illumination-independent observations, year-round monitoring, while maintaining accuracy levels comparable to optical system even under adverse atmospheric conditions. Among the evaluated classifiers, Random Forest (RF) showed the highest robustness and generalization capacity. Similarly, Banerjee et al. [17] classified crops using a random forest with an overall accuracy of 93% and 94%, followed by a Kappa coefficient of 0.9 and 0.89 in the two particular Indian locals. These results reinforce that SAR backscatter effectively captures phenological changes in rice under varying hydrological environment. The strong performance of RF algorithm can be attributed to its ensemble averaging and inherent resistance to noise and data imbalance, aligning with findings from Imangholiloo et al. [20] and Medina et al. [21], who also observed RF’s reliability in diverse smallholder landscapes.

The Support Vector Machine (SVM) model, on the other hand, showed higher omission errors, which were probably caused by kernel overfitting under imbalanced class distributions. While the CART and k-Nearest Neighbour (k-NN) classifier performed well for Aus, was less consistent for Aman reflecting greater intra-class variability caused by flooding and mixed land use. Overall, our study confirms that combining Sentinel-1 SAR with ML classifiers specifically, Random Forest and CART offer a reliable and adaptable framework for Aman and Aus respectively for cloud-resilient rice mapping in monsoon-prone areas. RF outperformed due to its ensemble learning capability, which reduces variance and improves generalization in heterogeneous landscapes. CART performed better for Aus due to simpler class separability (Rice, Non-Rice) and lower intra-class variability during that season. Although k-NN achieved higher pixel-level accuracy, it tends to overfit local patterns and is sensitive to class imbalance, leading to over-classification of rice pixels. This results in inflated or inconsistent area estimates compared to RF, which generalizes better due to ensemble averaging.

## 6. Limitations

In this study, a very few ground-truth samples were considered for Aus and Aman, which would have affected the calibration of the classifier. Field sampling across underrepresented regions should be expanded in future studies. Furthermore, the analysis covered one cropping year. Multi-year validation is needed to assess the inter-annual stability of model performance under varying climatic conditions. Deep-learning architectures could improve temporal feature extraction for multi-year rice dynamics, even though RF did the best in this case.

A key limitation of the evaluation scheme relates to sample extraction and spatial autocorrelation. Reference polygons were converted into pixel samples using the Google Earth Engine sampleRegions() function, and the train/test split (80/20) was executed at the pixel level rather than at the polygon level. Because neighboring pixels from the same field polygon share near-identical SAR backscatter characteristics, spatial autocorrelation introduced spatial data leakage between training and testing sets. This artificially inflated the pixel-level overall classification accuracies. In addition, no explicit class balancing techniques (e.g., class weighting, oversampling, or undersampling) were applied during model training. Although independent validation and cross-validation indicated stable model performance, future studies will investigate balanced sampling and cost-sensitive learning approaches to further reduce potential classification bias. Although deep learning approaches (e.g., LSTM, CNN) have shown improved capability in capturing temporal dependencies, their implementation within the Google Earth Engine is currently limited due to lack of native support for model training. Therefore, this study focuses on classical machine learning models that are computationally efficient and directly deployable within GEE for large-scale applications. No explicit cropland mask was applied; however, Non-Rice classes were included in training samples to allow the model to learn discrimination.

## 7. Conclusion

The suggested framework defined the spatial extent of both major rice seasons, Kharif-I and Kharif-II, and validated the results using official agricultural statistics. CART slightly outperformed other classifiers for Aus (overall accuracy = 99.6%), while the RF model performed the most consistent performance for Aman, with results that nearly matched BBS statistics. The suggested methods dependability was confirmed by the estimated Aman area, which was only 0.01% greater than the official BBS figures, confirming the reliability of the proposed approach. In case of Aus, 28% deviation was observed. Comparable accuracy levels (>90%) have been reported in earlier studies [4], further supporting the robustness of Sentinel-1 data for large-scale rice mapping in monsoon-prone regions. Through automated preprocessing, the GEE environment provides extra benefits by enabling effective, consistent data management across wide spatial and temporal domains. When taken as a whole, these characteristics show that a scalable, functional process for near-real-time rice monitoring in cloud-prone tropical locations is feasible.

Future work should extend this framework through multi-year validation, Sentinel-1/2 data fusion to enhance class separability. Integration of the rice area results with process-based crop models as well as DSSAT may be used for yield forecasting. The proposed methodology establishes a strong foundation for climate-resilient agricultural monitoring, supporting early-warning systems, disaster assessment, and sustainable food-security planning in Bangladesh and other monsoon-dominated rice-growing regions.
